# Supplementary material for: Analysis of Synonymous Codon Usage Bias in Potato Virus M and Its Adaption to Hosts
Source: Viruses. 2019 Aug 14;11(8):752. doi: 10.3390/v11080752 (PMC6722529; doi:10.3390/v11080752)
Supplement: Supplementary file 1 [file viruses-11-00752-s001.zip › Table S1-The PVM using in this study-190312.docx]

Table S1, The PVM isolates using in this study.

| Country | Isolate | Time | Host | Accession number | | Reference |
| --- | --- | --- | --- | --- | --- | --- |
|  |  |  |  | CP | NABP |  |
| Bangladesh | 171 | 2015 | *Potato* | MG365509 | MG365509 |  |
| Bangladesh | 165 | 2015 | *Potato* | MG365509 | MG365508 |  |
| Canada | Ca513 | 2006 | *Solanum tuberosum* | EF063389 | NA |  |
| Canada | Ca508 | 2006 | *Solanum tuberosum* | EF063388 | NA |  |
| Canada | Ca128 | 2006 | *Solanum tuberosum* | EF063387 | NA |  |
| Canada | Ca5 | 2006 | *Solanum tuberosum* | EF063386 | NA |  |
| Canada | CL4 | 2006 | *Solanum tuberosum* | EF063385 | NA |  |
| Canada | CL3 | 2006 | *Solanum tuberosum* | EF063384 | NA |  |
| Canada | CL1 | 2006 | *Solanum tuberosum* | EF063383 | NA |  |
| China | YN | 2016 | *Potato* | KY364848 | KY364848 | Sun et al., 2017 |
| China | YN-6-4 | 2011.1 | *Solanum muricatum* | KF561668 | KF561668 | Ge et al., 2014 |
| China | YN-1-7 | 2011.1 | *Solanum muricatum* | KF561667 | KF561667 | Ge et al., 2014 |
| China | YN-1-2 | 2011.1 | *Solanum muricatum* | KF561666 | KF561666 | Ge et al., 2014 |
| China | GS-6-6 | 2010.07 | *Solanum muricatum* | KF561665 | KF561665 | Ge et al., 2014 |
| China | GS-6-5 | 2010.07 | *Solanum muricatum* | KF561664 | KF561664 | Ge et al., 2014 |
| China | GS-T-10 | 2010.07 | *Lycopersicon esculentum* | KF561663 | KF561663 | Ge et al., 2014 |
| China | GS-T-8 | 2010.07 | *Lycopersicon esculentum* | KF561662 | KF561662 | Ge et al., 2014 |
| China | GS-T-1 | 2010.07 | *Lycopersicon esculentum* | KF561661 | KF561661 | Ge et al., 2014 |
| China | YN-6-3 | 2011.1 | *Solanum muricatum* | KF561660 | KF56166 | Ge et al., 2014 |
| China | YN-6-1 | 2011.1 | *Solanum muricatum* | KF561659 | KF561659 | Ge et al., 2014 |
| China | YN-5-1 | 2011.1 | *Solanum muricatum* | KF561658 | KF561658 | Ge et al., 2014 |
| China | YN-4-7 | 2011.1 | *Solanum muricatum* | KF561657 | KF561657 | Ge et al., 2014 |
| China | YN-4-2 | 2011.1 | *Solanum muricatum* | KF561656 | KF56165 | Ge et al., 2014 |
| China | YN-4-1 | 2011.1 | *Solanum muricatum* | KF561655 | KF561655 | Ge et al., 2014 |
| China | YN-3-7 | 2011.1 | *Solanum muricatum* | KF561654 | KF561654 | Ge et al., 2014 |
| China | YN-3-6 | 2011.1 | *Solanum muricatum* | KF561653 | KF561653 | Ge et al., 2014 |
| China | YN-3-5 | 2011.1 | *Solanum muricatum* | KF561652 | KF561652 | Ge et al., 2014 |
| China | YN-3-4 | 2011.1 | *Solanum muricatum* | KF561651 | KF561651 | Ge et al., 2014 |
| China | YN-3-2 | 2011.1 | *Solanum muricatum* | KF561650 | KF561650 | Ge et al., 2014 |
| China | YN-2-10 | 2011.1 | *Solanum muricatum* | KF561649 | KF561649 | Ge et al., 2014 |
| China | YN-2-9 | 2011.1 | *Solanum muricatum* | KF561648 | KF561648 | Ge et al., 2014 |
| China | YN-2-6 | 2011.1 | *Solanum muricatum* | KF561647 | KF561647 | Ge et al., 2014 |
| China | YN-1-9 | 2011.1 | *Solanum muricatum* | KF561646 | KF561646 | Ge et al., 2014 |
| China | YN-1-8 | 2011.1 | *Solanum muricatum* | KF561645 | KF561645 | Ge et al., 2014 |
| China | YN-1-6 | 2011.1 | *Solanum muricatum* | KF561644 | KF561644 | Ge et al., 2014 |
| China | YN-1-5 | 2011.1 | *Solanum muricatum* | KF561643 | KF561643 | Ge et al., 2014 |
| China | YN-1-1 | 2011.1 | *Solanum muricatum* | KF561642 | KF561642 | Ge et al., 2014 |
| China | GS-6-7 | 2010.07 | *Solanum muricatum* | KF561641 | KF561641 | Ge et al., 2014 |
| China | GS-6-4 | 2010.07 | *Solanum muricatum* | KF561640 | KF561640 | Ge et al., 2014 |
| China | GS-6-3 | 2010.07 | *Solanum muricatum* | KF561639 | KF561639 | Ge et al., 2014 |
| China | GS-6-2 | 2010.07 | *Solanum muricatum* | KF561638 | KF561638 | Ge et al., 2014 |
| China | GS-6-1 | 2010.07 | *Solanum muricatum* | KF561637 | KF561637 | Ge et al., 2014 |
| China | GS-5-7 | 2010.07 | *Solanum muricatum* | KF561636 | KF561636 | Ge et al., 2014 |
| China | GS-4-5 | 2010.07 | *Solanum muricatum* | KF561635 | KF561635 | Ge et al., 2014 |
| China | GS-4-1 | 2010.07 | *Solanum muricatum* | KF561634 | KF561634 | Ge et al., 2014 |
| China | GS-3-8 | 2010.07 | *Solanum muricatum* | KF561633 | KF561633 | Ge et al., 2014 |
| China | GS-3-7 | 2010.07 | *Solanum muricatum* | KF561632 | KF561632 | Ge et al., 2014 |
| China | GS-3-6 | 2010.07 | *Solanum muricatum* | KF561631 | KF561631 | Ge et al., 2014 |
| China | GS-3-3 | 2010.07 | *Solanum muricatum* | KF561630 | KF561630 | Ge et al., 2014 |
| China | GS-2-8 | 2010.07 | *Solanum muricatum* | KF561629 | KF561629 | Ge et al., 2014 |
| China | GS-2-6 | 2010.07 | *Solanum muricatum* | KF561628 | KF561628 | Ge et al., 2014 |
| China | GS-2-4 | 2010.07 | *Solanum muricatum* | KF561627 | KF561627 | Ge et al., 2014 |
| China | GS-2-3 | 2010.07 | *Solanum muricatum* | KF561626 | KF561626 | Ge et al., 2014 |
| China | GS-2-2 | 2010.07 | *Solanum muricatum* | KF561625 | KF561625 | Ge et al., 2014 |
| China | GS-2-1 | 2010.07 | *Solanum muricatum* | KF561624 | KF561624 | Ge et al., 2014 |
| China | GS-1-5 | 2010.07 | *Solanum muricatum* | KF561623 | KF561623 | Ge et al., 2014 |
| China | GS-1-4 | 2010.07 | *Solanum muricatum* | KF561622 | KF561622 | Ge et al., 2014 |
| China | GS-1-2 | 2010.07 | *Solanum muricatum* | KF561621 | KF561621 | Ge et al., 2014 |
| China | JL-4-10 | 2011.1 | *Solanum muricatum* | KF561620 | KF561620 | Ge et al., 2014 |
| China | JL-4-9 | 2011.1 | *Solanum muricatum* | KF561619 | KF561619 | Ge et al., 2014 |
| China | JL-4-7 | 2011.1 | *Solanum muricatum* | KF561618 | KF561618 | Ge et al., 2014 |
| China | JL-4-6 | 2011.1 | *Solanum muricatum* | KF561617 | KF561617 | Ge et al., 2014 |
| China | JL-4-5 | 2011.1 | *Solanum muricatum* | KF561616 | KF561616 | Ge et al., 2014 |
| China | JL-4-4 | 2011.1 | *Solanum muricatum* | KF561615 | KF561615 | Ge et al., 2014 |
| China | JL-4-3 | 2011.1 | *Solanum muricatum* | KF561614 | KF561614 | Ge et al., 2014 |
| China | JL-4-1 | 2011.1 | *Solanum muricatum* | KF561613 | KF561613 | Ge et al., 2014 |
| China | JL-3-9 | 2011.1 | *Solanum muricatum* | KF561612 | KF561612 | Ge et al., 2014 |
| China | JL-3-8 | 2011.1 | *Solanum muricatum* | KF561611 | KF561611 | Ge et al., 2014 |
| China | JL-3-7 | 2011.1 | *Solanum muricatum* | KF561610 | KF561610 | Ge et al., 2014 |
| China | JL-3-3 | 2011.1 | *Solanum muricatum* | KF561609 | KF561609 | Ge et al., 2014 |
| China | JL-3-4 | 2011.1 | *Solanum muricatum* | KF561608 | KF561608 | Ge et al., 2014 |
| China | JL-3-5 | 2011.1 | *Solanum muricatum* | KF561607 | KF561607 | Ge et al., 2014 |
| China | JL-3-6 | 2011.1 | *Solanum muricatum* | KF561606 | KF561606 | Ge et al., 2014 |
| China | JL-2-10 | 2011.1 | *Solanum muricatum* | KF561605 | KF561605 | Ge et al., 2014 |
| China | JL-2-9 | 2011.1 | *Solanum muricatum* | KF561604 | KF561604 | Ge et al., 2014 |
| China | JL-2-7 | 2011.1 | *Solanum muricatum* | KF561603 | KF561603 | Ge et al., 2014 |
| China | JL-2-6 | 2011.1 | *Solanum muricatum* | KF561602 | KF561602 | Ge et al., 2014 |
| China | JL-2-4 | 2011.1 | *Solanum muricatum* | KF561601 | KF561601 | Ge et al., 2014 |
| China | JL-2-5 | 2011.1 | *Solanum muricatum* | KF561600 | KF561600 | Ge et al., 2014 |
| China | JL-2-3 | 2011.1 | *Solanum muricatum* | KF561599 | KF561599 | Ge et al., 2014 |
| China | JL-2-2 | 2011.1 | *Solanum muricatum* | KF561598 | KF561598 | Ge et al., 2014 |
| China | JL-2-1 | 2011.1 | *Solanum muricatum* | KF561597 | KF561597 | Ge et al., 2014 |
| China | JL-1-8 | 2011.1 | *Solanum muricatum* | KF561596 | KF561596 | Ge et al., 2014 |
| China | JL-1-4 | 2011.1 | *Solanum muricatum* | KF561595 | KF561595 | Ge et al., 2014 |
| China | JL-1-3 | 2011.1 | *Solanum muricatum* | KF561594 | KF561594 | Ge et al., 2014 |
| China | JL-1-2 | 2011.1 | *Solanum muricatum* | KF561593 | KF561593 | Ge et al., 2014 |
| China | JL-1-1 | 2011.1 | *Solanum muricatum* | KF561592 | KF561592 | Ge et al., 2014 |
| China | QH-2-5 | 2011.05 | *Solanum muricatum* | KF561591 | KF561591 | Ge et al., 2014 |
| China | QH-2-1 | 2011.05 | *Solanum muricatum* | KF561590 | KF561590 | Ge et al., 2014 |
| China | QH-1-11 | 2011.05 | *Solanum muricatum* | KF561589 | KF561589 | Ge et al., 2014 |
| China | QH-1-8 | 2011.05 | *Solanum muricatum* | KF561588 | KF561588 | Ge et al., 2014 |
| China | QH-1-6 | 2011.05 | *Solanum muricatum* | KF561587 | KF561587 | Ge et al., 2014 |
| China | QH-1-4 | 2011.05 | *Solanum muricatum* | KF561586 | KF561586 | Ge et al., 2014 |
| China | BJ-1-9 | 2011.04 | *Solanum muricatum* | KF561585 | KF561585 | Ge et al., 2014 |
| China | BJ-1-6 | 2011.04 | *Solanum muricatum* | KF561584 | KF561584 | Ge et al., 2014 |
| China | BJ-1-4 | 2011.04 | *Solanum muricatum* | KF561583 | KF561583 | Ge et al., 2014 |
| China | BJ-1-1 | 2011.04 | *Solanum muricatum* | KF561582 | KF561582 | Ge et al., 2014 |
| China | Gansu | 2011.06 | *Lycopersicon esculentum* | JN835299 | JN835299 | Ge et al., 2012 |
| China | Hangzhou | 2002 | *Solanum muricatum* | AJ437481 | AJ437481 |  |
| China | M1 | 2011.07 | *Solanum tuberosum* | KF408265 | NA |  |
| Czech Republic | VIRUBRA 4/007 | 1985 | *Solanum tuberosum* | HM854296 | HM854296 | |
| Czech Republic | VIRUBRA 4/009 | 1980 | *Solanum tuberosum* | JN225461 | JN225461 |  |
| Czech Republic | VIRUBRA 4/016 | 1993 | *Solanum tuberosum* | HM991708 | HM991708 | |
| Czech Republic | VIRUBRA 4/035 | 1999 | *Solanum tuberosum* | HQ005276 | HQ005276 | |
| German | German isolate | 1990 | *Lycopersicon esculentum* | X57440 | X57440 | Gramstat et al.,1990 |
| Germany | DSMZ PV0273 | 2008 | *Nicotiana tabacum* | EU604672 | EU604672 | Flatken et al.,2008 |
| Hungary | 20810384 | 2008 | *Solanum tuberosum* | GQ923785 | NA |  |
| India | Jau-33 | 2013.02 | *Solanum tuberosum* | KJ473993 | KJ473993 |  |
| India | Gaj-13 | 2013.12 | *Solanum tuberosum* | KJ473992 | KJ473992 |  |
| India | PVM-Del-144 | 2013.01 | *Solanum tuberosum* | KJ194171 | KJ194171 |  |
| India | HAT-12 | 2013.12 | *Solanum tuberosum* | KJ919966 | NA |  |
| India | KAN-16 | 2013.12 | *Solanum tuberosum* | KJ919965 | NA |  |
| India | AGF-5 | 2013.12 | *Solanum tuberosum* | KJ919964 | NA |  |
| India | Mat-12 | 2013.02 | *Solanum tuberosum* | KJ569697 | KJ569697 |  |
| India | PVM-Del-133 | 2013.02 | *Solanum tuberosum* | KJ569696 | KJ569696 |  |
| India | Del-147 | 2013.02 | *Solanum tuberosum* | KJ462137 | NA |  |
| India | Del-134 | 2013.12 | *Solanum tuberosum* | KJ462136 | NA |  |
| India | Del-123 | 2013.03 | *Solanum tuberosum* | KJ462135 | NA |  |
| India | Mir-12 | 2013.12 | *Solanum tuberosum* | KJ462134 | NA |  |
| India | Bal-21 | 2013.02 | *Solanum tuberosum* | KJ462133 | NA |  |
| India | M34 | 2012.09 | *Solanum tuberosum* | KF471070 | NA |  |
| Iran | 352 | 2011 | *Potato* | JX678982 | JX678982 |  |
| Iran | 739 | 2011.07 | *solanum tuberosum* | NA | KC699728 | Tabasinejad et al., 2015 |
| Iran | 691 | 2011.06 | *solanum tuberosum* | NA | KC699727 | Tabasinejad et al., 2015 |
| Iran | 689 | 2011.06 | *solanum tuberosum* | NA | KC699726 | Tabasinejad et al., 2015 |
| Iran | 655 | 2011.06 | *solanum tuberosum* | NA | KC699725 | Tabasinejad et al., 2015 |
| Iran | 511 | 2011.05 | *solanum tuberosum* | NA | KC699724 | Tabasinejad et al., 2015 |
| Iran | 501 | 2011.05 | *solanum tuberosum* | NA | KC699723 | Tabasinejad et al., 2015 |
| Iran | 492 | 2011.05 | *solanum tuberosum* | NA | KC699722 | Tabasinejad et al., 2015 |
| Iran | 490 | 2011.05 | *solanum tuberosum* | NA | KC699721 | Tabasinejad et al., 2015 |
| Iran | 448 | 2011.05 | *solanum tuberosum* | NA | KC699720 | Tabasinejad et al., 2015 |
| Iran | 380 | 2011.06 | *solanum tuberosum* | NA | KC699719 | Tabasinejad et al., 2015 |
| Iran | 375 | 2011.06 | *solanum tuberosum* | NA | KC699718 | Tabasinejad et al., 2015 |
| Iran | 371 | 2011.05 | *solanum tuberosum* | NA | KC699717 | Tabasinejad et al., 2015 |
| Iran | 369 | 2011.07 | *solanum tuberosum* | NA | KC699716 | Tabasinejad et al., 2015 |
| Iran | 352 | 2011.06 | *solanum tuberosum* | NA | KC699715 | Tabasinejad et al., 2015 |
| Iran | 286 | 2011.06 | *solanum tuberosum* | NA | KC699714 | Tabasinejad et al., 2015 |
| Iran | 264 | 2011.06 | *solanum tuberosum* | NA | KC699713 | Tabasinejad et al., 2015 |
| Iran | 352 | 2011.07 | *Solanum tuberosum* | JX678982 | JX678982 |  |
| Iran | 758 | 2011.06 | *Solanum tuberosum* | KC479343 | NA | Tabasinejad et al., 2014 |
| Iran | 749 | 2011.06 | *Solanum tuberosum* | KC479342 | NA | Tabasinejad et al., 2014 |
| Iran | 745 | 2011.06 | *Solanum tuberosum* | KC479341 | NA | Tabasinejad et al., 2014 |
| Iran | 739 | 2011.06 | *Solanum tuberosum* | KC479340 | NA | Tabasinejad et al., 2014 |
| Iran | 706 | 2011.06 | *Solanum tuberosum* | KC479339 | NA | Tabasinejad et al., 2014 |
| Iran | 691 | 2011.06 | *Solanum tuberosum* | KC479338 | NA | Tabasinejad et al., 2014 |
| Iran | 656 | 2011.06 | *Solanum tuberosum* | KC479337 | NA | Tabasinejad et al., 2014 |
| Iran | 655 | 2011.06 | *Solanum tuberosum* | KC479336 | NA | Tabasinejad et al., 2014 |
| Iran | 515 | 2011.05 | *Solanum tuberosum* | KC479335 | NA | Tabasinejad et al., 2014 |
| Iran | 511 | 2011.05 | *Solanum tuberosum* | KC479334 | NA | Tabasinejad et al., 2014 |
| Iran | 453 | 2011.05 | *Solanum tuberosum* | KC479333 | NA | Tabasinejad et al., 2014 |
| Iran | 439 | 2011.05 | *Solanum tuberosum* | KC479332 | NA | Tabasinejad et al., 2014 |
| Iran | 380 | 2011.05 | *Solanum tuberosum* | KC479331 | NA | Tabasinejad et al., 2014 |
| Iran | 315 | 2011.06 | *Solanum tuberosum* | KC479330 | NA | Tabasinejad et al., 2014 |
| Iran | 760 | 2011.06 | *Solanum tuberosum* | KC129101 | NA | Tabasinejad et al., 2014 |
| Iran | 734 | 2011.06 | *Solanum tuberosum* | KC129100 | NA | Tabasinejad et al., 2014 |
| Iran | 732 | 2011.06 | *Solanum tuberosum* | KC129099 | NA | Tabasinejad et al., 2014 |
| Iran | 689 | 2011.06 | *Solanum tuberosum* | KC129098 | NA | Tabasinejad et al., 2014 |
| Iran | 688 | 2011.06 | *Solanum tuberosum* | KC129097 | NA | Tabasinejad et al., 2014 |
| Iran | 519 | 2011.05 | *Solanum tuberosum* | KC129096 | NA | Tabasinejad et al., 2014 |
| Iran | 501 | 2011.05 | *Solanum tuberosum* | KC129095 | NA | Tabasinejad et al., 2014 |
| Iran | 490 | 2011.05 | *Solanum tuberosum* | KC129094 | NA | Tabasinejad et al., 2014 |
| Iran | 448 | 2011.05 | *Solanum tuberosum* | KC129093 | NA | Tabasinejad et al., 2014 |
| Iran | 371 | 2011.05 | *Solanum tuberosum* | KC129092 | NA | Tabasinejad et al., 2014 |
| Iran | 369 | 2011.07 | *Solanum tuberosum* | KC129091 | NA | Tabasinejad et al., 2014 |
| Iran | 352 | 2011.06 | *Solanum tuberosum* | KC129090 | NA | Tabasinejad et al., 2014 |
| Iran | 310 | 2011.06 | *Solanum tuberosum* | KC129089 | NA | Tabasinejad et al., 2014 |
| Iran | 291 | 2011.06 | *Solanum tuberosum* | KC129088 | NA | Tabasinejad et al., 2014 |
| Iran | 286 | 2011.06 | *Solanum tuberosum* | KC129087 | NA | Tabasinejad et al., 2014 |
| Iran | 264 | 2011.06 | *Solanum tuberosum* | KC129086 | NA | Tabasinejad et al., 2014 |
| Latvia | La | 2007.9 | *Solanum tuberosum* | GQ496609 | NA |  |
| Poland | M57 | 2003 | *Unknown* | AY311395 | AY311395 |  |
| Poland | Uran | 2003 | *Unknown* | AY311394 | AY311394 |  |
| Russian | Russian wild | 2008 | *Lycopersicon esculentum* | D14449 | D14449 | Rupasov et al.,1989 |
| Russian | Russian wild | 2008 | *Lycopersicon esculentum* | NC_001361 | NC_001361 | Zavriev et al., 1991 |
| Slovakia | T40 | 2018 | *tomato* | MH558037 | MH558037 |  |
| Slovakia | T50 | 2018 | *tomato* | MH558036 | MH558036 |  |
| Slovakia | T20 | 2018 | *tomato* | MH558053 | MH558053 |  |
| Tanzania | TZ:PVM12U:11 | 2011.12 | *Solanum tuberosum* | KC866622 | NA |  |
| USA | Idaho | 1997 | *Unknown* | AF023877 | NA |  |
