# Supplementary material for: Analysis of Synonymous Codon Usage Bias in Potato Virus M and Its Adaption to Hosts
Source: Viruses. 2019 Aug 14;11(8):752. doi: 10.3390/v11080752 (PMC6722529; doi:10.3390/v11080752)
Supplement: Supplementary file 1 [file viruses-11-00752-s001.zip › viruses-559046-suppl-figures caption.pdf]

Supplementary material

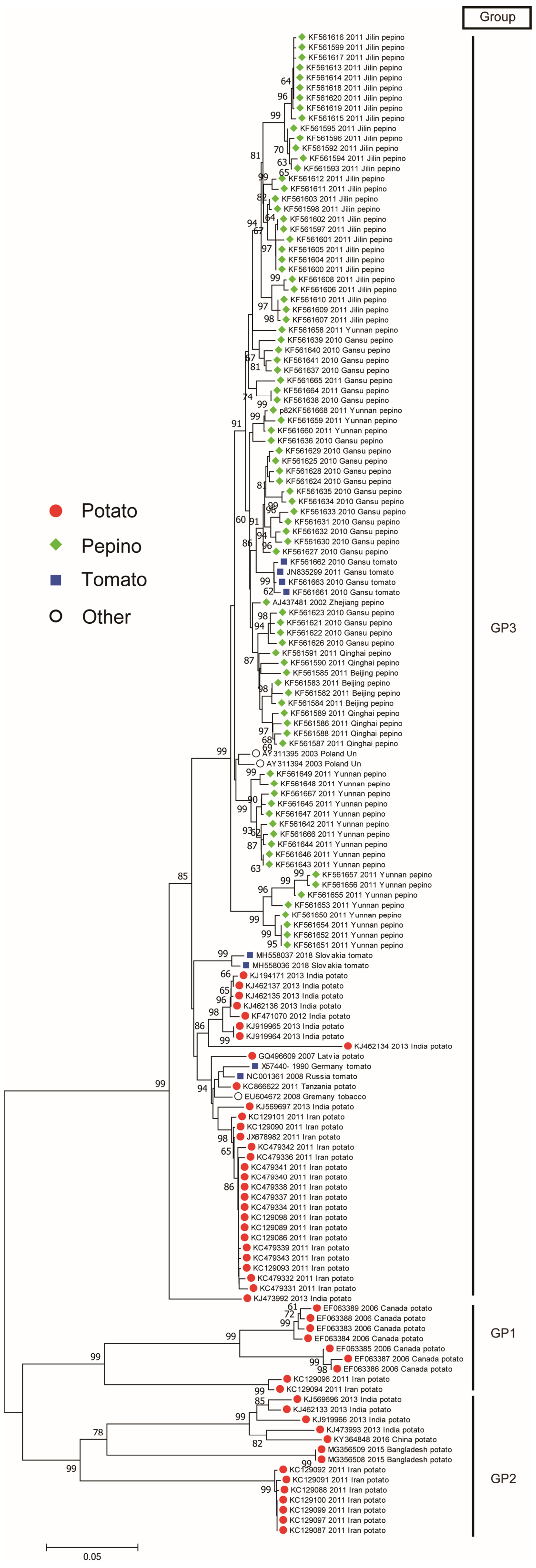

Figure S1. The neighbor-joining (NJ) trees calculated from the CP gene sequences of potato virus M obtained in this study. The numbers at each node indicate the percentage of bootstrap samples in the NJ trees. Horizontal branch length is drawn to scale, with the bar indicating 0.05 nt replacements per site.

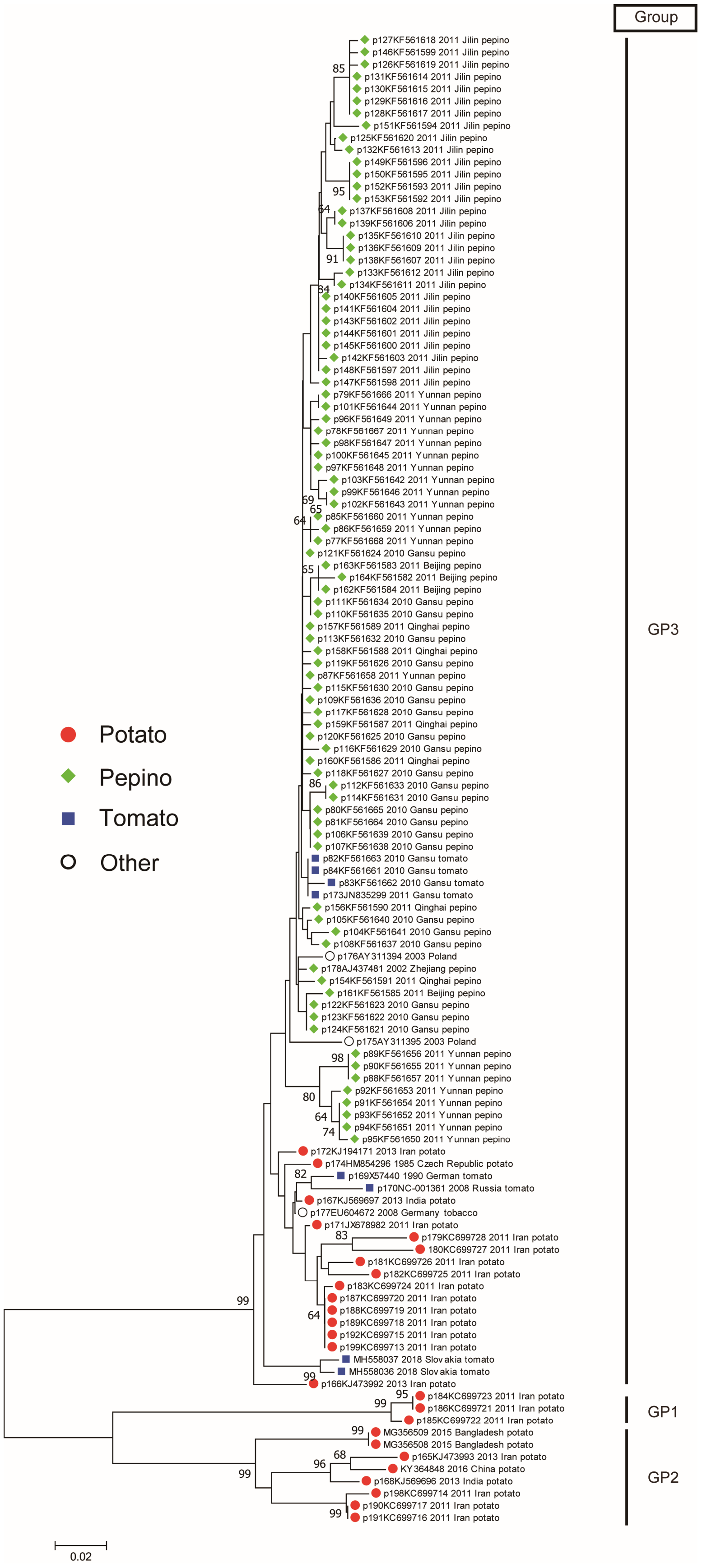

Figure S2. The neighbor-joining (NJ) trees calculated from the NABP gene sequences of potato virus M obtained in this study. The numbers at each node indicate the percentage of bootstrap samples in the NJ trees. Horizontal branch length is drawn to scale, with the bar indicating 0.02 nt replacements per site.
